# *Some* Remarks *on the* Origin *and* Progress *of the* Malignant Yellow Fever, *as It Appeared in the Village of Catskill, State of New-York, during the Summer and Autumn of* 1803: *In a Letter from Dr.* Benjamin W. Dwight *to* Eneas Monson, *M. D. of New-Haven, Connecticut*

**Published:** 1805-10-01

**Authors:** 


*Some* REMARKS *on the* ORIGIN *and* PROGRESS *of the* MALIGNANT YELLOW FEVER, *as it appeared in the Village of Catskill, State of New-York, during the Summer and Autumn of* 1803 : *In a Letter from Dr.* BENJAMIN W. DWIGHT *to* ENEAS MONSON, *M. D. of New-Haven, Connecticut.*

[ Continued from Vol. xiii. pp. 304—320. ]

HAVING stated, as I trust, a sufficient number of cases to evince the nature of the epidemic at its commencement, I shall now proceed to mention the ordinary symptoms which occurred in patients generally, and the extraordinary symptoms which occurred in particular cases.

Previous to the onset of the disease, patients generally complained of slight indisposition, such as is usual before the attack of fever. This was quickly succeeded, in most instances, but not in all, by a chilly fit. In a short time violent pain came on in the fore-part of the head, in the eyes, loins, and hips, in many instances extending down the thighs to the knees. In some instances the pain was most severe about the knees, and in that part of the leg where the gastrocnemius and soleus muscles unite to form the tendo Achillis. The face was much flushed; the eyes, in some instances, were of a fiery red, in others of a dull red appearance, and very intolerant of light. There was a stinging or burning heat in the skin; the pulse was full, quick, and hard: the tongue covered with a whitish fur, and moist: the thirst, in some instances, was excessive, in others moderate: the stomach was distended and very irritable. The sick complained much of a burning heat, pain, and excessive sickness at the stomach. They were troubled with frequent retching, and occasionally with vomiting; but they seldom brought up any thing, in this stage of the disease, except mucus and their drinks, and a small quantity of a greenish and very acrid matter, which, at times, almost corroded the fauces. A great quantity of of gas was eructated.* The bowels were generally costive. Great restlessness and despondence, and frequent moaning, and tossing about the bed, to find, if possible, some relief from change of posture, were common symptoms. Several patients, who had formerly been affected with intermitting and remitting fevers, declared that the pain which they suffered from this disease was more severe than all that they had endured in their lives before. Upon going into a patient's room, the common salutation was, "I shall die, I cannot get well again." After a short period had elapsed, the stomach was the part most affected.

When stools were procured by injections or cathartics, they were usually thin, and not to any considerable degree bilious. Except the colouring which they acquired from calomel, they were whitish.

No distinct remissions or exacerbations occurred in any instance,† but the disease marched steadily on till it was arrested by medicine, or till it had finished its course.

The first stage of the disease generally lasted from one to three days. In a few instances, perhaps, it extended into the fourth day. About this time a yellowness of the eyes came on in many, but not in all instances. The pain was much lessened. The pulse became frequent, but not hard, nor very full. The skin was dry, but much less hot. If the patient became evidently better at the first stage, the recovery was, in some instances, very rapid; but if the disease was undermining the constitution, the uneasiness at the stomach and the restlessness increased, and stupor rapidly came on. Silly delirium was a frequent symptom in this stage of the disease. The yellowness became more and more manifest, and diffused over the face, and, in some instances, over the body. Suppression of urine, black-vomit, coma, convulsions, and coldness of the extremities, followed in rapid succession, and death soon closed the miserable scene.

These

* The quantity of gas discharged from the stomach and bowels was in some instances, so great as to induce a belief that it was owing either to a decomposition of the gastric liquor, or that it was a morbid secretion from the areolæ which secrete that liquor.

† The difference in this respect between yellow fever and the common bilious remitting fever, as it appeared in the neighbourhood, was very remarkable, and, as it seemed to me, the most characteristic symptom by which to distinguish the two diseases. We had several severe cases of bilious remitting fever within a few miles of the village, and in every instance there were distinct alternate remissions and exacerbations, so that the most inattentive by-standers noticed it.

These were the more ordinary appearances which the disease assumed. In one patient, a young lady, during the first stage of the disease, the hands and arms were nearly covered with large crimson-coloured blotches, many of which were round, and of the size of a half-dime; others were of the size and figure of a damascene.

During the second stage, hæmorrhages, in several instances, took place from the nose, mouth, and uterus.—W. Hammond, a day labourer, a man of a robust habit, had the disease severely. The symptoms were very unfavourable till they were cut short by a salivation. About three days after the soreness of the mouth came on, the gums became exceedingly spongy, and he discharged from his mouth, in the course of forty-eight hours, more than six pounds of florid blood. After this, the hæmorrhage ceased, and he recovered in a manner surprisingly rapid.

Hæmorrhages from the mouth, nose, anus, &c. were unusually frequent this year, in the common bilious remitting fever.

One patient, an Irishman, of a very vigorous habit, was, on the third day of his disease, in fine spirits, chatted, and enjoyed himself to a very unusual degree. He complained of no pain: his countenance was clear; his pulse nearly natural. About twelve o'clock he sat up in the bed and shaved himself. In the evening he was affected with continual hiccough. During the night he was affected with black-vomit to a great degree. Early the next morning he got up to the window, and hallooed to some persons, who were about ten rods distant, to bring him some meat and drink, and said he was very hungry. He laid down again, and complained of vast uneasiness at the stomach; and, after making one struggle, instantly expired.

Another patient was in the same cheerful mood, and, within two hours, was over powered with stupor to so great a degree, that it was very difficult to arouse him. He died not long after with black-vomit and convulsions. The cheerfulness in these two cases resembled that which is produced by drinking freely of wine or brandy. The first of these patients, as there was strong reason to suspect, had indulged himself in that way.

The last of these patients, and one other, had, during the last stage, frequent stools, resembling strong beef-brine, and highly fœtid.

One patient, Lavinia Parsons, who resided in the same house and room with Briggs several days, during the early part of his illness, becoming unwell, alarmed at her situation, ation, removed to Mr. Ferguson's, a distant and healthy part of the village. Continuing unwell, though not confined to her bed, she was, a few days after, taken in a carriage to Freehold, about 20 miles westward of Catskill. At Freehold she soon became more seriously sick, and was brought back to Ferguson's. Her disease was considered by Dr. Croswell as a very decided case of Typhus gravior. The fever lasted four or five weeks without any intermission. I saw her a few times during her sickness, and had the same opinion of its nature. Just before her death, being near at hand, I was sent for in great haste, and found her vomiting matter which very strongly resembled chocolate grounds.

A little girl, sister to Lavinia Parsons, who came from Connecticut in the month of August, went to Briggs's house, and staid there one night. This was soon after he was taken sick. The next morning she went to Ferguson's. About the same time that Lavinia was seized she began to complain. Her's was a very clearly marked case of *typhus gravior.* A few days after Lavinia died, she began to recover. From these facts may we not infer that the jail and yellow fever are species of the same genus? and that the miasmata producing yellow fever are not specific, but may, according to circumstances, produce jail, or yellow, or some other fever?

I will only add, that no other persons were affected with fever in this neighbourhood.

### TREATMENT.

Dr. Croswell and myself practised together considerably, and our mode of practice was similar. If called at the onset of the disease, after a little experience had taught us what type it had assumed, we made very liberal evacuations from the bowels. This was effected by calomel alone, or conjoined with jalap. Ten or twelve grains of each were usually given to an adult patient. This was frequently aided or preceded by stimulating injections. In some instances blood-letting was indispensable. To allay the excessive sickness and burning at the stomach, we tried, in several instances, the effervescing draught, but it was of no kind of service. Neither did the aq. ammon. acet. appear to alleviate those troublesome symptoms. Alkalies alone were of any real benefit, and a very liberal use of them was adopted. After the cathartic had ceased operating, calomel was administered in small and frequent doses, with or without opium, according to circumstances. Mercurial ointment was also, in a number of of instances, applied freely, so as, if possible, to ensure a salivation. While this plan was in operation, the bowels were kept free, by injections or otherwise. In a few instances we attempted to cure the disease by copious and long-continued sweating, but our success was not such as to warrant this mode of practice. It was with the utmost difficulty that sweating could be excited and kept up for any considerable length of time.

The drinks were toast-water, barley-water, rice-water, chicken-water, and good spruce beer; the last of which, in several instances, sat better upon the stomach than any thing else.

Particular pains were taken to keep the rooms of the sick well ventilated. Their linen was changed often: vinegar was sprinkled upon their floors, and its steam diffused about the room.

If the disease proceeded on to the second stage, and the irritability of the stomach increased, a large epispastic was applied to the abdomen, and sinapisms to the feet. If to these were added delirium, or comatose symptoms, epispastics to the neck, thighs, legs, and arms, were also used.

When patients *began* to convalesce, good old Madeira wine, and porter, were very serviceable. After a little period had elapsed, colombo and bark were used advantageously. But wine and porter were evidently preferable in the beginning. The elixir vitriol also was often useful.

Some persons were slow in recovering. Those whose systems became so far affected with mercury as to produce a soreness of the gums, all recovered, and generally in a very rapid manner.

No other fever occurred, during the prevalence of this disease, within the circle of its ravages.

The number of deaths, including Lavinia Parsons, was eight.

About forty persons were affected with the disease; perhaps I should say, more properly, that there were about thirty clearly marked cases of the disease, and as many as ten or twelve doubtful cases. The latter we considered, at the onset, as decided cases; for in most, or all such instances, the complaint commenced with striking symptoms of this disease, and generally threatened a violent attack. But the patients, instead of delaying twelve or eighteen hours, to ascertain whether they were about to be severely sick or not, applied immediately for medical aid. In such instances very free evacuations were procured from the bowels; after which sweating was, in several instances, excited, excited, and kept up for a considerable length of time. Thus the symptoms were relieved, and by the second or third day the patient was evidently on the recovery. Were I to express an opinion, I should say that these were undoubted cases of yellow fever, and that the disease was completely subdued in the first stage.

Of those who died, one person, who was upwards of fifty years of age, possessed a constitution much shattered with syphilis and hard drinking, and would not, probably, have survived an attack of any inflammatory fever; a second died from a relapse: a third, as there was strong reason to believe, from drinking rum when he was apparently out of danger: a fourth, Mrs. ——, who had reached the tenth day of the disease, and bid fair to recover, was seized with premature labour, and expired shortly after: two others had greatly fatigued themselves immediately previous to the onset of the disease; a seventh, who was seized very violently, did not apply for medical aid till the inflammatory symptoms had nearly subsided. The eighth was Lavinia Parsons.

When these things are taken into consideration, the disease, to say the least, was not more fatal here than it has heretofore been in New York, New Haven, Philadelphia, and various other towns.

Of those who were affected with the disease, one-third were attacked in August, and two-thirds in September. The last person that was attacked was seized September 28. Three died in August, and five in September. Females, in general, were handled much less severely than males. Six of those who died were males.

None of our patients, during or after convalescence, were, to any considerable degree, affected with œdematous swellings.

A number of patients, after convalescence, lost most or all of the hair from their heads.

I come next to make some observations with respect to the origin of the disease. In a former part of this letter, I remarked that the creek, about three-quarters of a mile from its mouth, took a turn to the west, and that, along this bend, a street was laid, running nearly at right angles with the main street. Around this corner, and south and west of it, a number of houses and stores are situated, nearly or directly upon the bank. To these houses, and a few others on the opposite sides of the streets, the disease was confined.

At the bottom of the bank is a narrow strip of made-ground, ground, on which are situated two or three stores. In one of these stores, during the month of May, between two and three hundred barrels of herrings were deposited. The brine, which is composed of the blood and oil of the fish, impregnated with salt, ran out, during the hot weather in July, in large quantities, on the floor. To conduct it off, a scuttle had been cut in the floor, and the floor was made descending from all quarters to this place. Of course, the brine settled upon the ground below, where it became highly putrid and offensive. The store has no cellar, and on one side no underpinning, so that any noxious effluvia which might be generated here would have abundant opportunity to escape and poison the surrounding atmosphere. From the nature and arrangement of the ground and buildings around (the bank forming nearly a semi-circle, and being from ten to fifteen, and in some places twenty feet higher than the ground on which the store stands, and being in a great measure, covered with buildings), it may be easily conceived, that those who lived in this neighbourhood must have daily and constantly inhaled a poison highly concentrated. Any effluvia existing in this place, unless possessing a specific gravity less than the atmospheric air, could not, without difficulty, escape far. The stench from this place was intolerable, as I often perceived, at the distance of several rods. To those who lived in its near neighbourhood it was less so, but by others it was much complained of. Some mechanics, who were employed from time to time in working in and about the store, complained of being much nauseated by the stench.*

Mr. H's house, that in which the disease commenced, is situated directly on the bank, about sixteen yards north of the store. The north end of the house stands on the top of the bank, the south end at the bottom. The family lived at the south end, on the lower floor, and nearly twenty feet lower than the surface of the street. Whenever the wind blew from the south it came directly from the shore upon them. The air here was also oftentimes damp, chilly, and confined. It may not be useless to

* October 16, I went, in company with several gentlemen, to the wharf on which this store stands. The sickness had entirely ceased, and all apprehensions of it had died away; but the stench was so unpleasant, notwithstanding the herrings had all been removed in August, and a considerable quantity of unslacked lime had been thrown in and under the store, that we retired in haste, without having staid our intended time.

to observe, further, that the room in which Mr. and Mrs. H. slept was situated under an open piazza, and that, after every rain during the latter part of July and the early part of August, it became damp, and oftentimes wet, so that the clothes hanging in the room were much moulded. Mr. H. had for several weeks previous to his sickness, been accustomed to open the store early in the morning, before breakfast, and to hoist the windows.

The three first persons who were affected with the disease lived in his house, and composed the whole of his family at this time. The two first were seized on the 10th of August: — the third on the 11th; — Briggs, the fourth, about the same time. He lived in a house situated on the bank, about ten yards distant. The fifth spent his days and nights principally in Mr. Croswell's printing office, which stands about four rods north of Mr. H's house. He was also not unfrequently in the near neighbourhood of the store. I cannot find the least shadow of a reason to suspect that either of the four first had been in any way exposed to *contagion.* The fifth patient, who was seized August 19th, was present at the interment of Mr. H. and hence some persons might conclude, took the disease from *contagion;* but let it be remembered that he was constantly encircled by an atmosphere highly *infectious.* The sixth, John M——, left New-York August 10, and arrived here the 11th, in the evening, about twelve hours after Briggs was taken sick. This man came up in the Commodore, a sloop plying between this and New-York. On her arrival she was immediately hauled up along side Day's wharf, which is adjoining that on which Hale's store stands. On board of the Commodore, John spent his nights, and around Hale's store he was employed during the day. Whether he took the disease here or in New-York is not *certain,* nor very material From several circumstances we are inclined to think he took it here. That he should have communicated it to any one is very improbable, as he died very soon, and as no one who visited him had the disease. The next cases occurred in such houses as were situated nearest the store.

To trace the progress of the disease minutely any further would be tedious and unnecessary. I shall only add, that it was confined to the near neighbourhood of this store; that every person who experienced an attack of it, except two or three, resided within a stone's throw of the store; that these were employed for a number of days, about their usual business, within two or three rods of the same place; place; and that none of the numerous visitors and watchers from other parts of the town were affected with the disease. Probably more than fifty persons visited Mr. and Mrs. H. during the first six days of their illness. They, and many others afterwards, visited and watched with the sick. Old and young, males, and not a small number of delicate females, p[illegible]formed for them these and other kind offices. Of course many persons, and some of them possessing habits in no small degree liable to be acted upon by noxious effluvia, were much exposed to contagion, if any existed; but none felt any ill effects from it. In no instance was there any good reason, in our opinion, to believe that the disease was communicated from one person to another.

That the cause which I have assigned will, by all persons, be deemed a satisfactory one, I have not the vanity to imagine. That it appeared so to us we have no hesitation in declaring. In confirmation of this opinion, the following fact may be mentioned. Saturday, August 13, Dr. Croswell and myself being desirous to explore the cause of the disease, examined the state of the store, and the ground beneath. Having spent some time in accomplishing this object, we stepped a few feet aside, and continued conversing together for near half an hour. Though we were to the windward of the store, and the wind blew fresh, I perceived, several times, the stench to be very unpleasant. During the remainder of the day I was affected with slight head-ach and some degree of nausea. The next morning I rode out four miles to visit a patient. In addition to the ill feelings of the preceding day, I soon began to be affected with a severe burning in the fauces and stomach, and a distressing pain in the fore-part of the head. These disagreeable feelings rapidly increased till I returned. In a short time after, a profuse evacuation from the bowels took place, and I became almost immediately relieved. For eight or ten days after this, my perspiration was of a very peculiar and offensive smell. The burning in the throat and stomach produced a sensation altogether different from severe heart-burn.

If the cause which I have assigned was not the true one, I may safely say that it remains yet to be discovered. No other cause which is capable of a moment's defence, has yet been suggested, at least within my knowledge. Most of the inhabitants acceded to the opinion of its being produced by the herring-brine. All acknowledged this to be *causa sine qua non.* Some persons, however, it is but but just to mention, attributed it to another and very different cause. Early in the morning of the 9th of August, Mr. H. and family, and various other persons, were called upon to see a negro woman who lived in the neighbourhood, and who was supposed by the family to be expiring. This woman had been sick for several weeks; and some persons, rather than admit the yellow fever to originate from so *inefficacious* a cause as putrid fish or brine, concluded that it must have been communicated from Mary, the negress above-mentioned. How, or where she acquired the disease, no one could tell, till fruitful conjecture at length unravelled the mystery:—A person possessing a very luxuriant imagination, and terrified at the name of yellow fever, some time about the middle of August, passed near the negro house, and observed a basket of clothes standing at a little distance from the door. He "thought they appeared dirty," and smelt unpleasantly, and cautioned all those within hearing, not to come too near, for, to use his own expression, he "guessed that the clothes did not contain any thing good, and that there was no advantage in standing too near them." After guessing a little more, some uneasiness was excited in the minds of the bystanders. This hint, so gratifying to popular prejudice, was industriously circulated, and gained colouring and strength from every mouth that delights in telling some new thing; and it was soon currently reported, that Harry, Mary's husband, who was employed as a sailor on board the sloop Commodore, had purchased a chest of clothes in New-York, at auction; that these clothes had, in all probability, belonged to some person who had died of the yellow fever; and that in this way the disease was undoubtedly communicated to his wife, and from her to others. This was declared by Harry, in the most solemn manner, to be wholly without foundation. His wife, and mother (who is a very sober woman), declared that he had not brought home any articles of clothing, at any time during the present season, except such as he usually carried with him. Capt. Post, master of the Commodore, whose character for veracity and uprightness, and vigilant attention to his business, needs no commendation, also assured me repeatedly, that Harry had not, at any time during the present season, brought up any articles of clothing, except such as he usually carried with him, nor any boxes, nor trunks, nor chests, nor baskets, nor any thing else of the like kind. All this did not satisfy such as were unwilling to be satisfied. But after a few days had elapsed, the subject was cleared up to our entire satisfaction.

On Friday, the 12th of August, when the Commodore, which had arrived from New-York the evening before, was unlading her cargo, a basket of clothes belonging to a negro passenger, was brought to Harry's House, and placed a little distance from the door. While standing here the man above-mentioned espied it. This was that baleful fountain whence issued such exhalations as filled all the surrounding atmosphere with poison and death. Let it be remembered that the vessel which brought this basket of clothes did not arrive here till August 11th, about twelve hours after Briggs (the fourth case) was taken sick, and not till near a month after Mary was seized with her complaint. In what manner this basket of clothes introduced the yellow fever, it requires more ingenuity than I possess to determine. In addition to all this, Mary, the negro woman, had not the yellow fever, neither has she had it at any time during the present season. About the middle of July she was attacked with common bilious remitting fever; a fever which *was* wholly different, in its beginning and progress, from the yellow fever. It came on very slowly, she gradually becoming sick for near a week before she was confined to her bed, and it was marked daily with distinct alternate remissions and exacerbations. This cannot be said of the yellow fever, *as it appeared here*, in any instance. At the time Mr. H. and family, and others, were called upon to witness her apprehended departure, she had a violent hysteric fit, a complaint to which she was much snbject. She had at this time been convalescing for more than a week. The idea that any person should have caught the yellow fever from her in this situation is truly laughable, especially when we consider that none of her numerous watchers, so far as can be ascertained, had the disease.

This long, and I fear, tedious statement, might have been omitted, had I not deemed it advisable to strip the subject bare of that false and darkening veil which has been cast over it.

No other mode of accounting for the existence of the disease has yet been suggested, within my knowledge. Mrs. H. who is a very domestic lady, had been but a few rods from the house for several weeks. Mr. H. Emma, and Briggs, had not, so far as can be ascertained, been in any way exposed to contagion. The irresistible conclusion is, that the disease originated here; and where, let me me ask, is the improbability of such an event? Does not the climate of the United States, aided by filth and other local causes, in some part or other of it, give birth to diseases, every year, of as malignant a nature as the yellow fever? A very malignant dysentery, you well know, has appeared this season in New-Haven and Stratford, and many other parts of New-England, which has been more fatal than the yellow fever at Catskill. The last year, the towns of Northampton, Greenfield, and Blandford, in Massachusetts, suffered very severely from a most malignant epidemic; yet no person supposes that the disease, in any of these towns, was imported from a foreign country. Nearly one twenty-third part of all the inhabitants, as I was informed by Dr. Hunt, died in Northampton, in the year 1802; a town usually uncommonly healthy.

Were physicians, in all parts of our country, careful to write accurate histories of the epidemics which fall under their observation, I cannot but think that the doubts on the domestic origin of the yellow fever would be in a great measure removed.

The foregoing observations I have detailed to you with that freedom which ought to be exercised in discussing any interesting subject; confident that, if I am in an error, you will exercise towards me all that candour and friendship which I have heretofore experienced from your hands; and that if, on the contrary, I have laboured in supporting a just cause, you will not be the less ready to admit the weight of facts, though they oppose the opinions which you have espoused.

